# Artificial sounds following biological rules: A novel approach for non-verbal communication in HRI

**DOI:** 10.1038/s41598-020-63504-8

**Published:** 2020-04-27

**Authors:** Beáta Korcsok, Tamás Faragó, Bence Ferdinandy, Ádám Miklósi, Péter Korondi, Márta Gácsi

**Affiliations:** 1 Department of Mechatronics, Optics and Mechanical Engineering Informatics, Faculty of Mechanical Engineering, Budapest University of Technology and Economics, Budapest, Hungary; 2 Department of Ethology, Eötvös Loránd University, Budapest, Hungary; 3 MTA-ELTE Comparative Ethology Research Group, Budapest, Hungary

**Keywords:** Animal behaviour, Biological models, Biotechnology

## Abstract

Emotionally expressive non-verbal vocalizations can play a major role in human-robot interactions. Humans can assess the intensity and emotional valence of animal vocalizations based on simple acoustic features such as call length and fundamental frequency. These simple encoding rules are suggested to be general across terrestrial vertebrates. To test the degree of this generalizability, our aim was to synthesize a set of artificial sounds by systematically changing the call length and fundamental frequency, and examine how emotional valence and intensity is attributed to them by humans. Based on sine wave sounds, we generated sound samples in seven categories by increasing complexity via incorporating different characteristics of animal vocalizations. We used an online questionnaire to measure the perceived emotional valence and intensity of the sounds in a two-dimensional model of emotions. The results show that sounds with low fundamental frequency and shorter call lengths were considered to have a more positive valence, and samples with high fundamental frequency were rated as more intense across all categories, regardless of the sound complexity. We conclude that applying the basic rules of vocal emotion encoding can be a good starting point for the development of novel non-verbal vocalizations for artificial agents.

## Introduction

With the growing importance of social robots and other artificial agents, the development of adequate communication in Human-Robot and Human-Computer Interaction (HRI and HCI) is becoming imperative. A common approach in developing the communicational signals of social robots and other artificial agents is to base them on human communication^1^ e.g., on speech^2^ and human-specific gestures^3^. Human-like communication seems to be a natural way of interaction for social robots, as human languages can convey high complexity in sharing information^4^, and e.g., facial gestures can express a wide variety of affective states^5^. However, this approach is frequently undermined by technological limitations relating to the perceptive, cognitive, and motion skills implemented in the agent, which can become more obvious during the course of interaction, leading to disappointment^6,7^. Overt similarity can also cause aversion towards human-like robots (Uncanny Valley^8,9^). Furthermore, the proposed functions of specific robots do not always require the level of complexity found in human communication^6^, or their capabilities and functions are not in line with that of humans (e.g., no need for head-turning with 360° vision^9^, no morphological limitations in sound production). To avoid these issues, another approach is to consider HRI as interspecific interaction in which the artificial agent is regarded as a separate species, and only has to be equipped with a basic level of social competence and communicational skills that are aligned with its function^9^. In this framework formation of non-verbal communicational signals of artificial agents rely heavily on the foundations of biological signalling and are based on the behaviour of social animals. A plausible example for such a basis could be the dog (*Canis familiaris*), with which humans have an interspecific bond that is, in many aspects, functionally analogous to the relationship needed in HRI^6,9,10^.

Upholding this approach, features of non-verbal communication not only show common aspects across human cultures e.g., in facial expressions^11^ and non-verbal vocalizations^12^, but we can also find similarities with the communicational signals of non-human animals^13,14^, for a review see^15^. These similarities allow the use of communicational signals that are based on general rules observed across multiple taxa^16^ or on the behaviour of specific animal species, e.g., dogs^6,17^ in artificial agents.

In case of emotionally expressive vocal signals, the similarities between taxa emerge due to the evolutionarily conservative processes of sound production and vocal tract anatomy in terrestrial tetrapods^18^, making sound-based communicational signals more conservative than many other. This phenomenon is best described by the source-filter framework, connecting the physiological processes and anatomical structures to the acoustic features of vocalizations^19^. The source-filter framework also explains the physiological connection between the inner state of an animal and the related vocalizations^15^. Vocalizations are thought to have developed from involuntary sounds of exhalation (e.g., due to quick movements when escaping a predator) usually connected to specific inner states, which through ritualization can lead to communicative sounds^20^. These sounds contain acoustic features influenced by the original physiological changes related to inner states, e.g., the tenseness of respiratory muscles and stretching of vocal folds via laryngeal muscles is increased during high arousal, leading to increased call length and pitch^15,21^. Chimpanzee (*Pan troglodytes*) screams emitted during a severe attack from conspecifics are higher in frequency and longer than screams produced under less aggressive attacks^22^. Similarly, in baboons^23^ calls produced in high arousal contexts featured longer individual call lengths and higher average fundamental frequency (among other parameter changes).

Previous studies have proven that emotionally expressive human non-verbal vocalizations are easily recognizable across cultures^24^. Humans are also able to recognise animal vocalizations as emotionally expressive sounds^14^, and rate their valence and intensity similarly to humans’ based on acoustic parameters such as the fundamental frequency (*f*_0_) or call length (e.g., dogs^25^; domestic pigs (*Sus scrofa*)^26,27^). Animal and human vocalizations with higher fundamental frequencies are perceived as more intense, while vocalizations consisting of short calls are rated as more positive in valence^25,26^. Thus, fundamental frequency and call length can serve as acoustic cues for the listeners, informing them about the inner state of the vocalizing individual even in interspecies communication^28,29^. The aforementioned acoustic cues are consistent with the existence of simple coding rules of affective vocalizations that are shared in mammals and that are the result of homologous signal production and neural processing^30^. These simple coding rules, namely that higher fundamental frequency is connected to higher intensity and shorter call length is connected to positive valence, are substantiated by studies on multiple mammalian species (for reviews see^14,15,21,31^) and in connection with various acoustic parameters (e.g. low harmonic-to-noise ratio is connected to higher arousal in baboons^32^, dogs^25^ and bonnet macaques (*Macaca radiata*)^33^.

A comparative fMRI study conducted on humans and dogs^34^ has shown that the acoustic cues connected to the emotional valence of vocalizations are processed similarly in both species. Another study by Belin *et al*.^35^ showed that the cerebral response of human participants differed between hearing positive or negative valence vocalizations of rhesus monkeys (*Macaca mulatta*) and cats (*Felis catus*), and one brain region (the ventrolateral orbitofrontal cortex) showed greater activation to negative valence vocalizations from both animal species and also humans. These conserved neural systems might help to decode emotions from vocalizations and sounds, providing a basis for emotionally expressive cross-species communication^15^. As interaction with social robots and other artificial agents can be viewed as cross-species communication^9^, exploring the processes and behavioural expressions of emotional states in humans and non-human animals can advance the development of artificial communication systems.

The use of non-verbal acoustic features for emotion expression has been studied in HRI^36,37^. Yilmazyildiz *et al*.^38^ provided an extensive review of the research area of Semantic-Free Utterances (SFU), which includes the research of gibberish speech, e.g., creating affectively expressive speech with no semantic content via manipulating the vowel nuclei and consonant clusters of syllables of existing languages^39^; non-linguistic utterances and utterances based on musical theory, e.g., melodies comprised of synthesized notes with modification of multiple acoustic parameters (fundamental frequency, fundamental frequency range, pause length and ratio, envelope, pitch contour and musical major and minor modes)^40^, auditory icons (e.g., decreasing pitch representing a falling object^38^); and paralinguistic utterances, e.g., human laughter^41^.

A detailed review by Juslin and Laukka^42^ found that basic emotional categories are expressed similarly in human non-verbal emotion expressions as in musical performance, following the same acoustic cues, drawing further attention to the evolutionary background of acoustic emotion expression in mammals. Although multiple SFU studies investigate acoustic parameters that have biological backgrounds, most of the research is focused on signals derived from human communication or culture, and therefore draw from a higher-level system. We propose that establishing simple coding rules for the emotional effect of artificial sounds based on interspecies similarities of sound production and signal processing represents a more fundamental approach with a strong evolutionary basis, which can serve as a complementary general principle to other viewpoints.

As human and animal vocalizations are acoustically complex signals, we follow a systematic approach to reveal which parameters of the vocalizations contribute to basic coding rules, and whether other acoustic parameters affect them. The vocalizations of mammals contain characteristic acoustic parameters due to the similar processes of sound production, e.g., formants, which are generated when the source sound is filtered through the vocal tract, attenuating certain frequencies^31^. Conversely, vocalizations of artificial agents are not bound to morphological structures and can be freely adjusted to the function of the robot. However, biological features increase the similarities of the artificial agents to living beings, which is generally desirable in their interactions with humans^6,16^. While the fundamental frequency and call length can be modified according to the general coding rules found in animal vocalizations^14,25,26^ even in simple artificially generated sine-wave sounds that do not depict commonplace terrestrial mammal vocalizations, adding other parameters characteristic of animal vocalizations can increase their perceived animacy.

**Figure 1:**
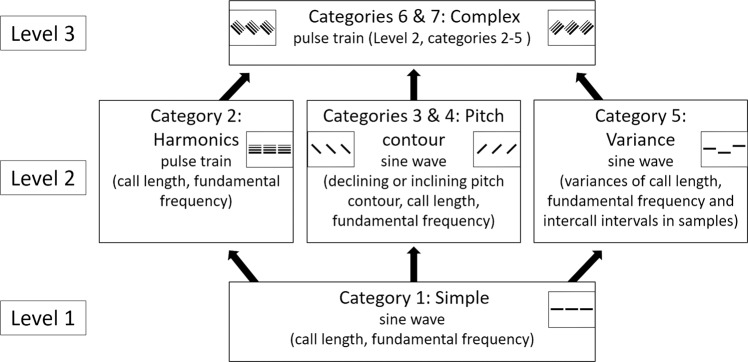
The categories of the artificial sounds across three levels of complexity. In each category the basis of the sound (sine wave or pulse train) is followed by the changed parameters in parenthesis. Level 1 category 1: Simple sine wave; Level 2 category 2: Pulse train; category 3: Sine wave sounds with pitch contour down; category 4: Sine wave sounds with pitch contour up; category 5: Variable sine wave; Level 3 category 6: Complex pulse train sounds with pitch contour down; category 7: Complex pulse train sounds with pitch contour up.

Following the previously outlined concepts, we created artificial sounds based on general acoustic features of emotionally expressive vocalisations of humans and non-human animals^25,31^. The specific ranges of the various acoustic parameters were mostly based on dog vocalizations, as these have been previously studied with a similar methodology to our own, providing an insight into how humans rate them on valence and intensity in a questionnaire study^25^, while we have less comparable results in e.g., primates. The sound samples were generated in multiple categories. We used sine-wave sounds for the simplest sound category, as these are single-frequency sounds that rarely occur naturally^43^, but which are frequently used in artificial signals of machines. Then, starting with the simple sine-wave sounds we added new acoustic features (pitch contour changes, harmonics, variations of call properties within a sound sample, formants) that are characteristic of animal vocalizations to make more complex and biologically more congruent samples. In each category, we systematically changed the fundamental frequency and call length of the sounds to cover the relevant acoustic ranges of these parameters (for more details see Fig. 1 and Table 1).

**Table 1:** Parameters of sound samples.

Parameters	Value or Range (across all samples)	Variance in categories 1.sin; 2.pulse; 3.pitch_d; 4.pitch_u	Variance in categories 5.var_sin; 6.comp_d; 7.comp_u	Reference
Fundamental frequency (*f*_0_)	65 Hz − 1365 Hz		uniformly distributed random value, ±5% of *f*_0_	~50–1600 Hz^25^
Total length (call length + interval length)	~2 s (+ silence until 3 s total duration)			2 s^25^
Call length	0.07; 0.16; 0.46; 0.76; 1.06; 1.96 s		uniformly distributed random value, ±25% of call length	0.11–2 s^25^
Intercall interval length	0.2 s	uniformly distributed random value, ±25% of interval length	uniformly distributed random value, ±50% of interval length	0.05–1.7 s^50^
Pitch contour change in categories 3.pitch_d; 4.pitch_u; 6.comp_d; 7.comp_u	uniformly distributed random value, ±10% of *f*_0_			^71^
Vocal tract length in categories 6.comp_d; 7.comp_u	20 cm			Modelling medium sized dog^72^
Number of formants in categories 6.comp_d; 7.comp_u	10			
First formant (f_1_) in categories 6.comp_d; 7.comp_u	550 Hz			

Categories: 1.sin: Simple sine wave; 2.pulse: Pulse train; 3.pitch_d: Sine wave sounds with pitch contour down; 4.pitch_u: Sine wave sounds with pitch contour up; 5.var_sin: Variable sine wave; 6.comp_d: Complex pulse train sounds with pitch contour down; 7.comp_u: Complex pulse train sounds with pitch contour up. More variance was implemented in the sounds of categories 5.var_sin, 6.com_d and 7.comp_u, than in the other categories. Pitch contour changes were only present in categories 3.pitch_d, 4.pitch_u, 6.comp_d and 7.comp_u, and formants were only modelled in the categories 6.comp_d and 7.comp_u.

## Questions and Hypotheses

Our main question was whether the simple coding rules of fundamental frequency and call length of vocalizations are also applied to artificially generated sounds.

Our hypothesis was:

H0: Simple coding rules do not exist, the direction of the effects of the acoustic parameters on the emotion ratings are different on the distinct complexity levels.

H1: Simple coding rules apply to artificial sounds as well, the direction of the effects of the acoustic parameters on the emotion ratings are the same.

In this latter case we expect that human listeners perceive artificial sounds with higher fundamental frequency as more intense and sounds with longer calls as having more negative valence, just like in case of human and animal sounds, as we can already find the simple coding rules in complex biological sounds evolved to communicate inner states. In parallel, neural systems are present to process these basic acoustic features. Moreover, if the presence of the features that are inherent consequences of the voice production system are inevitable for accepting a sound as biological and thus being a communicative signal encoding emotional states, the simple coding rules could have a stronger effect (a.k.a. stronger association between acoustic features and emotional scales) in more complex sounds.

## Method

### Subjects

All subjects were unpaid volunteers from various nationalities recruited via online advertisements. The number of participants in the final analysis were 237, from which 95 chose to fill the questionnaire in Hungarian (60 female, 35 male, mean age = 36.3 ± SD 11.8 years) and 142 in English (122 female, 20 male, mean age 39.9 ± SD 11.7 years). Questionnaire answers were discarded if the participant was under the age of 18. Part of our sample abandoned the survey before finishing (95 individuals) but as the sample presentation was random, these unfinished responses are unlikely to cause any bias, thus they were included in the analysis. Subjects gave their informed consent to participate in the study, which was carried out in accordance with the relevant guidelines and with the approval of the Institutional Review Board of the Institute of Biology, Eötvös Loránd University, Budapest, Hungary (reference number of the ethical permission: 2019/49).

### Stimuli

The artificial sounds were generated using a custom Praat (version 6.0.19) script (developed by TF and BK, see Supplementary Methods). The sound samples consisted of calls separated by mute intercall periods forming bouts. We varied both the lengths of the calls (cl) and the fundamental frequency (*f*_0_) in all cases. The range of most parameters was set in accordance with the non-verbal human and dog vocalizations used in^25^.

The range of fundamental frequency varied between 65 Hz to 1365 Hz with 100 Hz steps. There were multiple samples at each frequency step, with differing call lengths; sound samples were generated at every 0.03 s call length step. Following this, sounds with specific call lengths were selected (call length fell between 0.07 and 1.96 sec, for details see Table 1). The number of calls in a sound sample depended on the length of the calls, as the complete sound samples were consistently 3 s long and contained only complete calls, meaning that calls starting after 2 s, or calls that would have ended after the 3 s were muted, using Adobe Audition. Therefore, all sound samples consisted of a ~2 s part containing calls and ended with a ~1 s silent part. The intercall interval length was varied in all sound samples. The generated sounds showed variation in loudness, which we included in our analysis as a further acoustic parameter (mean loudness 79.4 ± SD 4.8 dB).

We created seven categories of artificial sounds with three levels of complexity. Figure 1 presents the characteristics of each category, while Table 1. shows a summary of the acoustic parameters of the sound samples.

Level 1 sounds (category 1) are based on sine waves in which only the call length and the fundamental frequency were varied. In Level 2 sounds (categories 2, 3, 4, 5) we systematically changed one aspect of the original simple sounds in each category. In category 2 we used pulse train sounds instead of sine waves, in which the consecutive non-sinusoid waves model the vibrations of the vocal folds, creating harmonics^19,44,45^. In categories 3 and 4 we implemented pitch contour changes with either decreasing or increasing pitch, while in the category 5 sounds we included variances in call length, in intercall interval length and in fundamental frequency (see Table 1). Level 3 sounds contained all the previously varied parameters (call length, fundamental frequency, harmonics, variances and pitch contour changes), as well as formants based on vocal tract modelling. The physical parameters of this model were defined as a hypothetical vocal tract for a ~70 cm tall social robot. The total number of created stimuli consisted of 588 sound samples, 84 in each category.

### Online questionnaire

The final questionnaire used in the study is accessible online at http://soundratingtwo.elte.hu. First, the participants were asked to provide demographic data on their nationality, gender and age, and were asked to answer the question whether they currently owned a dog at the time of the test or owned one in the past. The online page also provided the instructions for the questionnaire, explaining how to indicate the perceived valence and emotional intensity. The participants were asked to use headphones instead of loudspeakers to minimise the differences in the quality and the frequency range of sound production of built-in loudspeakers (e.g., laptops). Participants also had the opportunity to check if their headphones worked correctly and at an optimal volume by playing a non-relevant sound.

**Figure 2:**
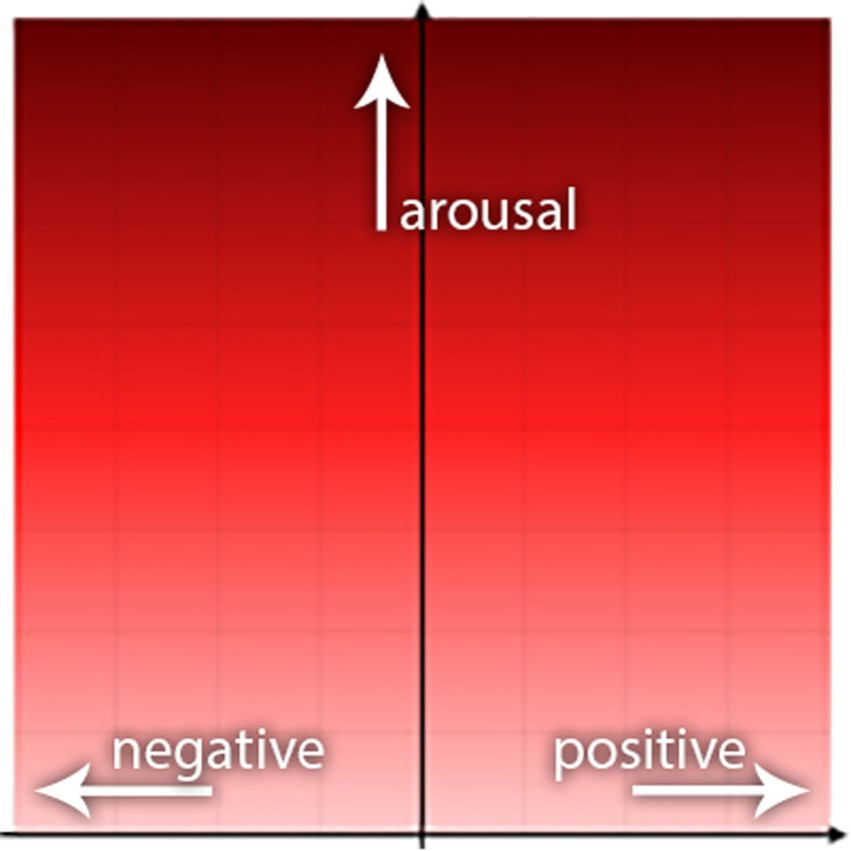
The intensity (scale from 0 to 100) and valence (scale from −50 to 50) axes of the questionnaire. Image first published in^25^.

The questionnaire used a modified version of the two-dimensional model of emotions by Russell^46^, which had already been successfully used for measuring the perceived emotions associated with dog and human vocalizations^25^. The questionnaire measured the values the participants gave for the sounds on the valence and intensity axes. We used the same questionnaire design in this study. After a sound was played, the participants had to indicate the valence on a horizontal axis and the intensity on the vertical one with one click (Fig. 2). Due to the high number of sound stimuli, each participant received only 11.9% of the samples (70 sound stimuli) after the 4 demo sounds, and received an equal number of samples (10) from all categories. The samples and their listening order were determined randomly.

### Data analysis

Statistical analysis was conducted in the R statistical environment.

We excluded responses slower than 20 seconds to avoid artefacts caused by network errors and possible lags in the stimuli presentation. Long response time might also indicate high uncertainty in the answer. We used Linear Mixed Modeling (lmer function from the lme4 package, version 1.1-21^47^) fit with backward elimination (drop1 function) to find the best model. The fixed effects were the fundamental frequency, call length, sound category, gender, age, query language (Hungarian or English), and the participants’ status of dog ownership, loudness of sound samples, as well as the two-way interactions of category and acoustic parameters; language, acoustic parameters and complexity category. The participant’s age, gender and dog ownership status were included as background variables, as these have been found to influence the perception of emotions in vocalizations in some cases^48–50^. The targets were the intensity and the valence values (respectively), and the random effects were the subjects and the ID of the sounds (see also in Table 2). We used a normal probability distribution with an identity link function and all covariates (fundamental frequency, call length, age, loudness) were scaled and centered. Loudness, and the interaction of loudness and category were included in the model after backward elimination. Tukey post-hoc tests (emmeans package, version 1.3.3^51^, emmeans and emtrends functions) were used for pairwise and trend comparisons.

**Table 2:** The linear mixed models used for statistical analysis. Cat: category, f0: fundamental frequency, cl: call length, age: age of the participant, lang: language of the query (English or Hungarian), dog: participants’ dog ownership status, gender: gender of the participant, loud: loudness of sound samples, testid: participant ID, soundid: ID of the sound samples.

		fixed effects	random effects
Intensity	intensity ~ cat + f0 + cl + age + lang + dog + gender + cat:f0 + cat:cl + lang:f0 + lang:cl + cat:lang + loud + cat:loud + (1|testid) + (1|soundid)	cat, f0, cl, age, lang, dog, gender, loud	testid, soundid
Valence	valence ~ cat + f0 + cl + age + lang + dog + gender + cat:f0 + cat:cl + lang:f0 + lang:cl + cat:lang + loud + cat:loud + (1|testid) + (1|soundid)	cat, f0, cl, age, lang, dog, gender, loud	testid, soundid

To compare the effects of call length and fundamental frequency in different complexity categories, we created a Linear Mixed Effects Model of category 1 (Simple sine wave), in which the fundamental frequency and the call length were fixed effects, subjects and sound ID were random effects, and the target was the valence or the intensity ratings. We used the models of category 1 to predict the valence and intensity ratings of the other categories. We compared the predicted and actual valence and intensity ratings with Pearson’s correlation.

## Results

### Intensity

We found that the simple Linear Mixed Effects Model fitted on the sinus category sounds predicted the intensity ratings of the other categories quite well based on the correlation between the real and predicted values (R = 0.49–0.60). The comparison of the predicted valence and intensity ratings of the categories is in Table 3.

**Table 3:** Comparison of predicted and actual ratings of valence and intensity. Predictive models are based on a Linear Mixed Effects Model of category 1 (Simple sine wave) sounds. 1.sin: Simple sine wave; 2.pulse: Pulse train; 3.pitch_d: Sine wave sounds with pitch contour down; 4.pitch_u: Sine wave sounds with pitch contour up; 5.var_sin: Variable sine wave; 6.comp_d: Complex pulse train sounds with pitch contour down; 7.comp_u: Complex pulse train sounds with pitch contour up, f0: fundamental frequency, cl: call length.

	Intensity				Valence			
Predictive model (based on 1.sin)		Est.	t	p		Est.	t	p
	Intercept	41.0812	31.8	<2.2e-16	Intercept	−8.2916	−7.121	<0.001
	f0	9.5927	16.2	<2.2e-16	f0	−5.4035	−7.945	<0.001
					cl	−3.2561	−4.777	<0.001
	r	df	p value	t	r	df	p value	t
1.sin	0.71	1670	<2.2e-16	41.268	0.70	1670	<2.2e-16	40.088
2.pulse	0.49	1690	<2.2e-16	23.345	0.46	1690	<2.2e-16	21.223
3.pitch_d	0.55	1672	<2.2e-16	26.595	0.46	1672	<2.2e-16	21.203
4.pitch_u	0.53	1657	<2.2e-16	25.225	0.53	1657	<2.2e-16	25.191
5.var_sin	0.60	1681	<2.2e-16	30.587	0.58	1681	<2.2e-16	29.015
6.comp_d	0.53	1675	<2.2e-16	25.465	0.47	1675	<2.2e-16	21.986
7.comp_u	0.50	1685	<2.2e-16	23.493	0.48	1685	<2.2e-16	22.491

**Figure 3:**
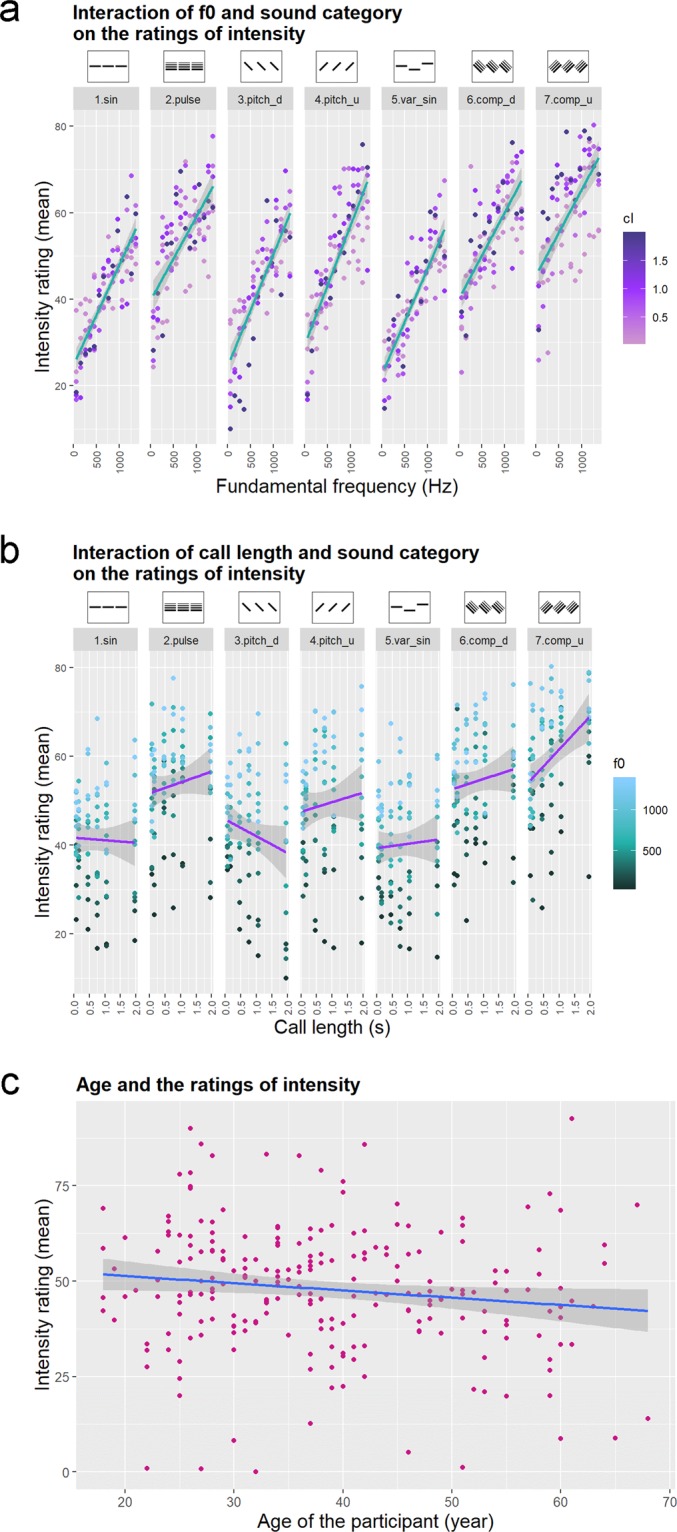
(**a**) The interaction of f0 and sound category on the ratings of intensity. Colouring of the dots shows the call length. (**b**) The interaction of call length and sound category on the ratings of intensity. Colouring of the dots shows the fundamental frequency. (**c**) The effect of the participants’ age on the ratings of intensity. Categories in (**a**) and (**b**): 1.sin: Simple sine wave; 2.pulse: Pulse train; 3.pitch_d: Sine wave sounds with pitch contour down; 4.pitch_u: Sine wave sounds with pitch contour up; 5.var_sin: Variable sine wave; 6.comp_d: Complex pulse train sounds with pitch contour down; 7.comp_u: Complex pulse train sounds with pitch contour up. The dots represent the mean intensity ratings of the sounds, while the grey shaded area around the regression line indicates the confidence interval at 95% confidence level.

In the Linear Mixed Model, both the fundamental frequency and call length were in interaction with the sound category and the language. According to the post-hoc tests, the fundamental frequency had a similar positive effect on intensity ratings in all categories: the sounds with higher fundamental frequency were rated as more intense, however this effect was stronger in category 4 (Sine wave up), 3 (Sine wave down), 5 (Variable sine wave) and 1 (Simple sine wave) while weaker in 2 (Pulse train), 6 and 7 (Complex pulse train down and up) (Fig. 3a). We see a similar pattern within both the English and the Hungarian responses, although stronger in the former. Call length had a negative effect in categories 1 (Simple sine wave) and 3 (Sine wave down): shorter calls were rated as more intense. In contrast, samples with longer calls were rated more intense in categories 2 (Pulse train), 4 (Sine wave up), 6 and 7 (Complex pulse train down and up) (Fig. 3b). In the Hungarian responses the post-hoc test showed a negative trend (short calls are more intense), compared to the English where the long calls were rated as more intense. The sound category was also in interaction with the language and loudness. In general English speaking respondents in most categories rated the samples as more intense compared to the Hungarian sample with the exception of categories 3 and 4 (Sine wave down and up) where we found no difference. In both languages categories 1 (Simple sine wave), 3 (Sine wave down) and 5 (Variable sine wave) got the lowest ratings, while 2 (Pulse train) the highest. Louder sounds were rated more intense in categories 2 (Pulse train), 7 (Complex pulse train up), 4 (Sine wave up), and 6 (Complex pulse train down). Age had a main effect, as older participants rated sounds as less intensive (Fig. 3c). The participants’ gender and dog-owner status had no effect on the intensity rating, thus were excluded from the final model. The results of the Linear Mixed Model are summarized in Table 4, and the post-hoc tests are summarized in Supplementary Tables S1 and S2.

**Table 4:** Results of the Linear Mixed Model fit of the intensity ratings. Pr(>F): the p-value belonging to the F statistics. Cat: category, f0: fundamental frequency, cl: call length, age: age of the participant, lang: language of the query, loud: loudness of sound samples.

	Sum Sq	Mean Sq	NumDF	DenDF	F value	Pr(>F)	
age	2274	2274.3	1	226.8	4.7418	0.030470	*
cat:f0	47594	7932.3	6	545.7	16.5385	<2.2e-16	***
cat:cl	10459	1743.2	6	547.2	3.6345	0.001515	**
f0:lang	4282	4282.1	1	11444.2	8.9279	0.002814	**
cl:lang	29122	29122.1	1	11447.5	60.7184	7.154e-15	***
cat:lang	26478	4413.0	6	11436.8	9.2009	4.462e-10	***
cat:loud	20891	3481.9	6	558.9	7.2595	1.763e-07	***

### Valence

We found that the simple Linear Mixed Effects Model fitted on the sinus category sounds predicted the valence ratings of the other categories quite well based on the correlation between the real and predicted values (R = 0.46–0.58). The comparison of the predicted valence and intensity ratings of the categories is in Table 3.

**Figure 4:**
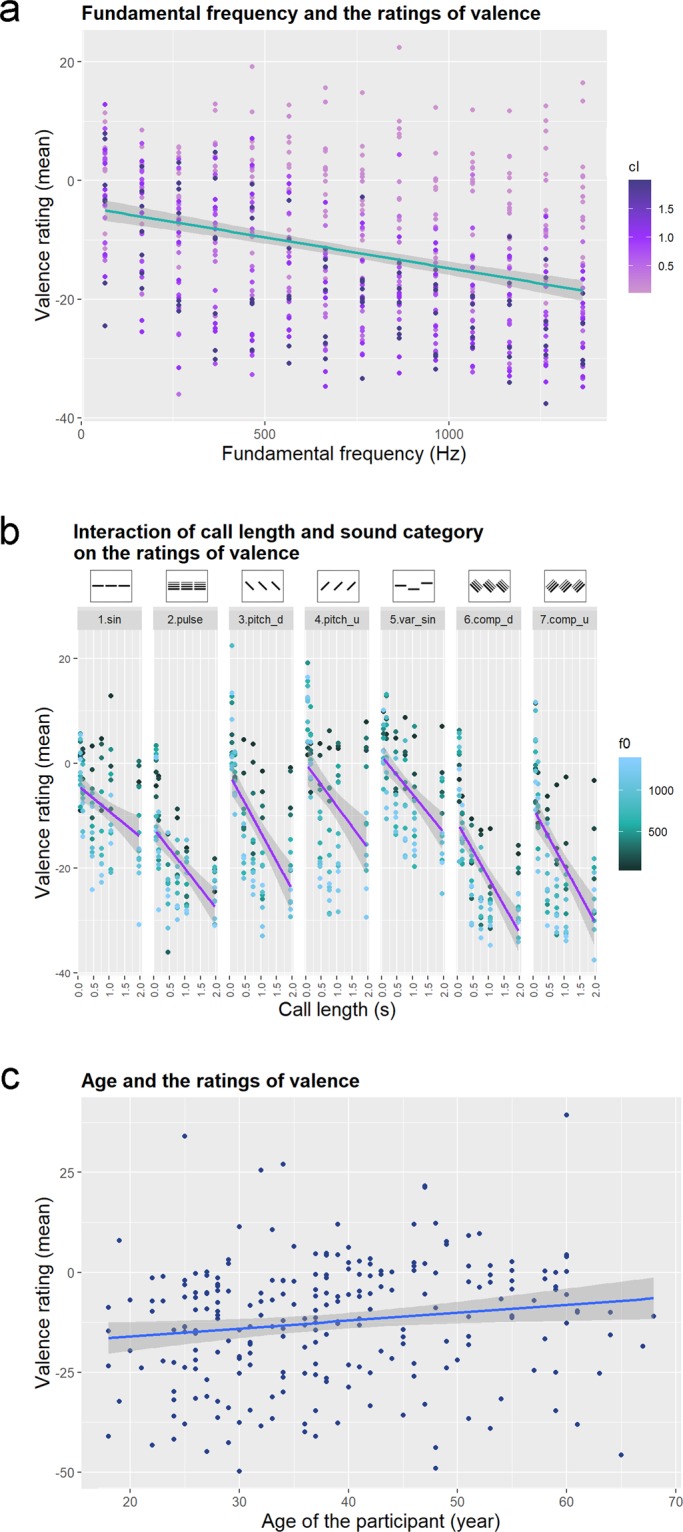
(**a**) The effect of fundamental frequency on the ratings of valence. Colouring of the dots shows the call length. (**b**) The interaction of call length and sound category on the ratings of valence. Colouring of the dots shows the fundamental frequency. (**c**) The effect of the participants’ age on the ratings of valence. Categories in (**b)**: 1.sin: Simple sine wave; 2.pulse: Pulse train; 3.pitch_d: Sine wave sounds with pitch contour down; 4.pitch_u: Sine wave sounds with pitch contour up; 5.var_sin: Variable sine wave; 6.comp_d: Complex pulse train sounds with pitch contour down; 7.comp_u: Complex pulse train sounds with pitch contour up. The dots represent the mean valence ratings of the sounds, while the grey shaded area around the regression line indicates the confidence interval at 95% confidence level.

The fundamental frequency had a significant main effect in the Linear Mixed Model: samples with lower fundamental frequency were rated to be more positive (Fig. 4a). The post hoc test showed that in the sound category and call length interaction the sound samples that consist of longer calls were rated as having a more negative valence in all categories (Fig. 4b). The interaction of sound category and language showed a significant language effect only within the 2nd (Pulse train) and 3rd (Sine wave down) category: Hungarian responses tended to be more positive in the former and more negative in the latter than English ratings. In both languages category 2 (Pulse train), 6 and 7 (Complex pulse train down and up) were the most negatively rated, while category 4 (Sine wave up) was the most positive. Louder sounds were generally rated as more negative, which effect was steepest in category 2 (Pulse train) and less so in categories 4 and 3 (Sine wave up and down). Age had a main effect: older participants rated the sounds as less negative regardless of the complexity category (Fig. 4c). The gender of the participants and their dog-owner status had no effect on the valence ratings, and neither did the interaction of language and call length, the interaction of language and fundamental frequency, and the interaction of complexity category and fundamental frequency. The results of the Linear Mixed Model are summarized in Table 5, and the post-hoc tests are summarized in Supplementary Tables S3 and S4.

**Table 5:** Results of the Linear Mixed Model fit of the valence ratings. Pr(>F): the p-value belonging to the F statistics. Cat: category, f0: fundamental frequency, cl: call length, age: age of the participant, lang: language of the query, loud: loudness of sound samples.

	Sum Sq	Mean Sq	NumDF	DenDF	F value	Pr(>F)	
f0	39154	39154	1	567.0	102.0804	<2.2e-16	***
age	3531	3531	1	220.0	9.2069	0.002701	**
cat:cl	15431	2572	6	569.3	6.7053	7.127e-07	***
cat:lang	21056	3509	6	11348.5	9.1494	5.150e-10	***
cat:loud	31721	5287	6	574.7	13.7834	1.155e-14	***

## Discussion

The results show that our artificially generated sounds are able to mimic some of the basic coding rules that are present in animal (mammalian) vocalizations. The predictive models based on sinus sound samples explain quite well both the valence and the intensity ratings in all other complexity categories suggesting the presence of the simple rules. The fundamental frequency of the sounds affects the perceived intensity, that is, sounds with higher fundamental frequency were perceived as more intense, while sounds containing longer calls were rated as more negative across all categories. These results align with the findings of previous research on animal and human vocalizations^14,25,26^.

An interesting result was the effect of fundamental frequency on valence: sounds with a higher fundamental frequency were rated as more negative in all categories. Although the fundamental frequency-valence effect was not found by Faragó *et al*.^25^ in dog or human vocalizations, the spectral centre of gravity showed a similar pattern in the case of human vocalizations. Multiple other studies also found that higher pitch was associated with negative valence, in e.g., dogs^50^, pigs^26^ and wild boars^52^, horses (*Equus caballus*)^53^ and bonobos (*Pan paniscus*)^54^. However, high frequency vocalizations in positive contexts can also be found (for a review see^31^), suggesting that the effect of pitch on valence might be non-linear, or can be influenced by other acoustic parameters.

Emotionally expressive vocalizations of terrestrial tetrapods are assumed to have evolved from involuntary sounds emitted due to breathing during aroused emotional states^55^. However, due to the morphological structures and processes of sound production, even simple emotionally expressive vocalizations are acoustically complex, e.g., phonation already appears in frog vocalizations with the appearance of vocal cords, and continues to be present in terrestrial mammals as a result of vocal fold or membrane vibration^56^. As the basic coding rules related to fundamental frequency and call length were also present in the artificial sounds with no added biological features, we can infer that these effects might originate from a more fundamental component of sound processing.

Communicational signals are frequently the result of ritualization, in which a behaviour that carries only involuntary information goes through an evolutionary process in which it becomes specialized and gains a signalling function^57,58^. Ritualization also increases signal complexity, leading e.g., to decreased signal ambiguity or to reproductive isolation via better species recognition^59^. Systematic investigations using generated sounds akin to ours could be used to find common aspects in the ritualized vocal signals of multiple species, aiding in the understanding of how evolutionary pressures affect specific acoustic parameters.

The results also underscore the compatibility of our approach with other SFU methods of emotion expression by showing that the added acoustic parameters did not interfere with the coding rules based on the acoustic cues derived from the call length and fundamental frequency. We found some overall differences in categories with pulse train sounds (categories 2, 6 and 7) as these were generally rated as more intense and more negative than the sounds in sine wave categories (categories 1, 3, 4 and 5). Pulse train sounds can be perceived to be noisier compared to sine wave sounds, which could have resulted in the higher intensity and more negative valence ratings. Furthermore, as pulse train sounds were used to approximate harmonics (category 2) and formants (categories 6 and 7) of animal and human vocalizations, these might have caused an unintended eeriness, which could have resulted in an uncanny effect (as described in HRI, e.g.^8,9^,) near fundamental frequencies that approximate human speech, leading to more intensive and negative ratings.

The call length of the sounds affected the intensity ratings differently in some of the categories, indicating that it does not represent a general coding rule. The effect of call length on intensity was not found in human vocalizations in^25^, only in dogs, which indicates that this association might be species-specific. By including other acoustic parameters in the artificial sounds, further systematic investigations could specify if some rules are species or taxon specific (e.g., in^25^ the tonality (harmonic-to-noise ratio, HNR) affected the intensity ratings of only dog vocalizations, as sounds with high HNR were rated as less intense) or if there are other general coding rules based on the added parameters. It could also clarify which parameters can be added to implement further rules with the potential to enrich or refine the range of expressible emotions.

Loudness influenced both the intensity and valence ratings in interaction with the categories: louder sounds were rated as more negative in all categories with varying degrees, while in case of intensity ratings the direction of the effect differed among the categories. As loudness of biological sounds is notoriously hard to measure reliably, especially in field recordings (recording distance and direction highly affects the measurements) this parameter cannot be compared between species, and its role in emotion encoding is uncertain. Although based on physiology and neural control of vocalization we can hypothesise that it can be linked with both higher arousal and negative inner states^31^. Our results partially support this, but it seems that fundamental frequency and call length plays a more crucial role in emotion encoding.

A limitation of the current set of sounds is the low number of sound samples that were rated notably positive. The majority of sounds had a mean rating on the valence axis lower than 0, and only a small number of sounds had a mean rating higher than 20. This presents a problem in the framework of human-robot interactions, as social robots are to exhibit behaviours also associated with positive emotions. However, considering the basis of these sounds, the scarcity of positive valence sounds is not surprising. In animal vocalisations, the expression of positive inner states is less frequent, and their functionality is limited to very specific behaviours or situations, e.g., grooming^60^, greeting^61^, play^62^. Vocalizations of dogs show a similar pattern in their perceived valence in contrast to human non-verbal vocalizations which cover the whole scale^25^. An acoustic parameter which is associated with positive inner states in humans is a steeper spectral slope^63^, which can be incorporated in the next iteration of the artificial sounds.

In some cases, the language of the questionnaire influenced the strength of the effects, and in the call length-intensity connection, its direction. As the effect of the call length on the intensity ratings was only present in interaction with the categories and not as a general rule independent of added acoustic parameters, it can be assumed that a slight difference of interpretation of the word ‘intensity’ by the Hungarian or English speaking participants could have caused this discrepancy. However, this seems to have no major confounding effect in the case of our main questions about the simple encoding rules.

We found that the age of the participant had a significant effect on both the valence and intensity ratings of the sounds, as older participants considered the sound samples to be more positive and less intensive than did younger adults. This could be explained by the neural changes that occur during ageing, which leads to a bias towards positive stimuli found in the elderly (positivity effect), causing increased attention towards^64^ and memories of^65^ positive stimuli. Elderly people are faster to recognize positive facial expressions than negative ones^66^, while studies have contradictory results on intensity ratings (increased intensity^66^; decreased intensity^67^). Age related hearing loss could have also influenced the answers of elderly participants, as hearing impairment is more prevalent in sounds with higher frequencies, starting from 1000 Hz^68,69^, which could somewhat reduce the effects of higher fundamental frequency on the intensity and valence ratings found on younger adults. However, the associations between the acoustic cues and the intensity and valence ratings persisted, despite the effects of age, and the noise caused by possible sound differences due to the headphone devices of the participants.

As the participants only rated the sounds on their intensity and valence, some functionally important aspects were not investigated. Based on the current results it is not possible to differentiate between sounds with high intensity and negative valence, as they may be perceived as ‘angry’ or ‘fearful’. However, vocalizations perceived as angry/aggressive or fearful/distressed usually elicit opposing behavioural responses from others, as the first may prompt behaviours to avoid the source of the sound, while fearful or distressed vocalizations may elicit approach^70^. This difference in the behavioural response to sounds is instrumental in HRI, and therefore should be investigated as an added dimension to the valence and intensity.

### Outlook

In the current study, we have established that humans assess the intensity and emotional valence of artificial sounds according to simple coding rules that are based on acoustic cues of animal vocalizations: sounds with higher fundamental frequency are perceived as more intense, while sounds with shorter call lengths are perceived as being more positive. As these coding rules are considered to be shared at least among mammals, the artificial sounds presumably elicit similar responses in non-human mammalian species that live in the human social environment. In our future work, we are planning on investigating the responses of humans and companion animals to the artificially generated sounds, with comparative fMRI studies on humans and dogs and with behavioural tests on humans, dogs and cats. We are also investigating the approach-avoidance responses of humans to the artificial sounds with a follow up questionnaire study.

Defining basic rules of emotion encoding using comparative approach can be the key to understanding the evolutionary processes of animal vocalizations. We suggest that the presented systematic method of assessing the effects of artificial sounds provides a novel opportunity to investigate the evolution of both the production and perception mechanisms underlying vocal emotion expression.

## Data Availability

The dataset generated during the current study is available as a supplementary file (Dataset.csv).

## Supplemantary information

Supplemantary information.
Supplemantary information 2.
Supplemantary information 3.
Supplemantary information 4.
Supplemantary information 5.
Supplemantary information 6.
Supplemantary information 7.
Supplemantary information 8.
Supplemantary information 9.
Supplemantary information 10.
Supplemantary information 11.
Supplemantary information 12.
