# Supplementary material for: Artificial sounds following biological rules: A novel approach for non-verbal communication in HRI
Source: Sci Rep. 2020 Apr 27;10:7080. doi: 10.1038/s41598-020-63504-8 (PMC7184580; doi:10.1038/s41598-020-63504-8)
Supplement: Supplementary file 11 — Supplemantary information 11. [file 41598_2020_63504_MOESM11_ESM.docx]

Supplementary results

**Artificial sounds following biological rules: A novel approach for non-verbal communication in HRI**

Beáta Korcsok^1*^, Tamás Faragó^2^, Bence Ferdinandy^3^, Ádám Miklósi^2,3^, Péter Korondi^1^, and Márta Gácsi^2,3^

*^1^Department of Mechatronics, Optics and Mechanical Engineering Informatics, Faculty of Mechanical Engineering, Budapest University of Technology and Economics, Budapest, Hungary*

*^2^Department of Ethology, Eötvös Loránd University, Budapest, Hungary*

*^3^MTA-ELTE Comparative Ethology Research Group, Budapest, Hungary*

*corresponding author: korcsok@mogi.bme.hu*

Intensity – Tukey post-hoc tests

Table S1

|  | categories | contrast | estimate | SE | df | z.ratio | p.value |
| --- | --- | --- | --- | --- | --- | --- | --- |
| lang\|cat | cat = 1 | English - Hungarian | 5.614 | 2.70 | Inf | 2.076 | 0.0379 |
|  | cat = 2 |  | 12.207 | 2.68 | Inf | 4.550 | <.0001 |
|  | cat = 3 |  | 2.259 | 2.72 | Inf | 0.829 | 0.4069 |
|  | cat = 4 |  | 0.022 | 2.73 | Inf | 0.008 | 0.9936 |
|  | cat = 5 |  | 6.279 | 2.71 | Inf | 2.317 | 0.0205 |
|  | cat = 6 |  | 7.604 | 2.71 | Inf | 2.810 | 0.0050 |
|  | cat = 7 |  | 9.307 | 2.71 | Inf | 3.434 | 0.0006 |
|  |  | contrast: categories | estimate | SE | df | z.ratio | p.value |
| cat\|lang | English | 1-2 | -30.4510 | 3.00 | Inf | -10.158 | <.0001 |
|  |  | 1-3 | 1.0698 | 1.45 | Inf | 0.736 | 0.9904 |
|  |  | 1-4 | -3.6779 | 1.46 | Inf | -2.523 | 0.1510 |
|  |  | 1-5 | 0.9749 | 1.43 | Inf | 0.681 | 0.9937 |
|  |  | 1-6 | -12.4837 | 1.27 | Inf | -9.825 | <.0001 |
|  |  | 1-7 | -17.6148 | 1.27 | Inf | -13.912 | <.0001 |
|  |  | 2-3 | 31.5207 | 2.99 | Inf | 10.538 | <.0001 |
|  |  | 2-4 | 26.7731 | 2.99 | Inf | 8.945 | <.0001 |
|  |  | 2-5 | 31.4259 | 2.98 | Inf | 10.541 | <.0001 |
|  |  | 2-6 | 17.9673 | 2.91 | Inf | 6.182 | <.0001 |
|  |  | 2-7 | 12.8361 | 2.90 | Inf | 4.419 | 0.0002 |
|  |  | 3-4 | -4.7477 | 1.44 | Inf | -3.287 | 0.0175 |
|  |  | 3-5 | -0.0948 | 1.42 | Inf | -0.067 | 1.0000 |
|  |  | 3-6 | -13.5535 | 1.25 | Inf | -10.808 | <.0001 |
|  |  | 3-7 | -18.6846 | 1.25 | Inf | -14.946 | <.0001 |
|  |  | 4-5 | 4.6529 | 1.42 | Inf | 3.269 | 0.0186 |
|  |  | 4-6 | -8.8058 | 1.26 | Inf | -6.989 | <.0001 |
|  |  | 4-7 | -13.9369 | 1.26 | Inf | -11.103 | <.0001 |
|  |  | 5-6 | -13.4587 | 1.23 | Inf | -10.951 | <.0001 |
|  |  | 5-7 | -18.5898 | 1.22 | Inf | -15.179 | <.0001 |
|  |  | 6-7 | -5.1311 | 1.03 | Inf | -4.977 | <.0001 |
|  | Hungarian | 1-2 | -23.8586 | 3.39 | Inf | -7.041 | <.0001 |
|  |  | 1-3 | -2.2860 | 2.11 | Inf | -1.082 | 0.9335 |
|  |  | 1-4 | -9.2702 | 2.11 | Inf | -4.397 | 0.0002 |
|  |  | 1-5 | 1.6400 | 2.08 | Inf | 0.789 | 0.9861 |
|  |  | 1-6 | -10.4941 | 1.96 | Inf | -5.353 | <.0001 |
|  |  | 1-7 | -13.9217 | 1.97 | Inf | -7.077 | <.0001 |
|  |  | 2-3 | 21.5727 | 3.39 | Inf | 6.359 | <.0001 |
|  |  | 2-4 | 14.5885 | 3.39 | Inf | 4.304 | 0.0003 |
|  |  | 2-5 | 25.4986 | 3.37 | Inf | 7.566 | <.0001 |
|  |  | 2-6 | 13.3646 | 3.30 | Inf | 4.049 | 0.0010 |
|  |  | 2-7 | 9.9369 | 3.30 | Inf | 3.008 | 0.0421 |
|  |  | 3-4 | -6.9842 | 2.11 | Inf | -3.308 | 0.0164 |
|  |  | 3-5 | 3.9259 | 2.08 | Inf | 1.889 | 0.4875 |
|  |  | 3-6 | -8.2081 | 1.96 | Inf | -4.182 | 0.0006 |
|  |  | 3-7 | -11.6358 | 1.97 | Inf | -5.911 | <.0001 |
|  |  | 4-5 | 10.9102 | 2.07 | Inf | 5.261 | <.0001 |
|  |  | 4-6 | -1.2239 | 1.96 | Inf | -0.625 | 0.9960 |
|  |  | 4-7 | -4.6515 | 1.96 | Inf | -2.369 | 0.2120 |
|  |  | 5-6 | -12.1340 | 1.93 | Inf | -6.303 | <.0001 |
|  |  | 5-7 | -15.5617 | 1.93 | Inf | -8.061 | <.0001 |
|  |  | 6-7 | -3.4277 | 1.80 | Inf | -1.899 | 0.4806 |
|  |  | contrast: categories | estimate | SE | df | z.ratio | p.value |
|  | cl:cat | 1-2 | 0.955 | 1.26 | Inf | 0.760 | 0.9886 |
|  |  | 1-3 | 3.835 | 1.31 | Inf | 2.930 | 0.0527 |
|  |  | 1-4 | 0.227 | 1.30 | Inf | 0.175 | 1.0000 |
|  |  | 1-5 | -1.305 | 1.30 | Inf | -1.004 | 0.9533 |
|  |  | 1-6 | -0.387 | 1.22 | Inf | -0.318 | 0.9999 |
|  |  | 1-7 | -1.094 | 1.24 | Inf | -0.881 | 0.9755 |
|  |  | 2-3 | 2.880 | 1.26 | Inf | 2.283 | 0.2524 |
|  |  | 2-4 | -0.728 | 1.25 | Inf | -0.582 | 0.9973 |
|  |  | 2-5 | -2.260 | 1.25 | Inf | -1.806 | 0.5436 |
|  |  | 2-6 | -1.342 | 1.17 | Inf | -1.149 | 0.9128 |
|  |  | 2-7 | -2.049 | 1.19 | Inf | -1.720 | 0.6025 |
|  |  | 3-4 | -3.608 | 1.30 | Inf | -2.769 | 0.0821 |
|  |  | 3-5 | -5.140 | 1.30 | Inf | -3.940 | 0.0016 |
|  |  | 3-6 | -4.222 | 1.22 | Inf | -3.450 | 0.0101 |
|  |  | 3-7 | -4.929 | 1.25 | Inf | -3.955 | 0.0015 |
|  |  | 4-5 | -1.532 | 1.29 | Inf | -1.184 | 0.9004 |
|  |  | 4-6 | -0.614 | 1.21 | Inf | -0.507 | 0.9988 |
|  |  | 4-7 | -1.321 | 1.24 | Inf | -1.069 | 0.9371 |
|  |  | 5-6 | 0.918 | 1.21 | Inf | 0.756 | 0.9889 |
|  |  | 5-7 | 0.211 | 1.24 | Inf | 0.171 | 1.0000 |
|  |  | 6-7 | -0.707 | 1.15 | Inf | -0.614 | 0.9964 |
|  |  | contrast: categories | estimate | SE | df | z.ratio | p.value |
|  | f0:cat | 1-2 | 8.546 | 1.396 | Inf | 6.123 | <.0001 |
|  |  | 1-3 | -0.973 | 0.929 | Inf | -1.047 | 0.9429 |
|  |  | 1-4 | -2.178 | 0.933 | Inf | -2.335 | 0.2273 |
|  |  | 1-5 | -0.269 | 0.933 | Inf | -0.288 | 1.0000 |
|  |  | 1-6 | 3.439 | 1.141 | Inf | 3.013 | 0.0413 |
|  |  | 1-7 | 4.982 | 1.134 | Inf | 4.394 | 0.0002 |
|  |  | 2-3 | -9.519 | 1.397 | Inf | -6.815 | <.0001 |
|  |  | 2-4 | -10.723 | 1.399 | Inf | -7.666 | <.0001 |
|  |  | 2-5 | -8.814 | 1.399 | Inf | -6.300 | <.0001 |
|  |  | 2-6 | -5.106 | 1.546 | Inf | -3.304 | 0.0166 |
|  |  | 2-7 | -3.563 | 1.540 | Inf | -2.314 | 0.2369 |
|  |  | 3-4 | -1.205 | 0.932 | Inf | -1.292 | 0.8559 |
|  |  | 3-5 | 0.705 | 0.933 | Inf | 0.755 | 0.9890 |
|  |  | 3-6 | 4.413 | 1.141 | Inf | 3.867 | 0.0021 |
|  |  | 3-7 | 5.955 | 1.134 | Inf | 5.250 | <.0001 |
|  |  | 4-5 | 1.909 | 0.937 | Inf | 2.038 | 0.3908 |
|  |  | 4-6 | 5.617 | 1.144 | Inf | 4.912 | <.0001 |
|  |  | 4-7 | 7.160 | 1.136 | Inf | 6.304 | <.0001 |
|  |  | 5-6 | 3.708 | 1.145 | Inf | 3.239 | 0.0205 |
|  |  | 5-7 | 5.251 | 1.137 | Inf | 4.617 | 0.0001 |
|  |  | 6-7 | 1.543 | 1.314 | Inf | 1.175 | 0.9039 |
|  |  | contrast: categories | estimate | SE | df | z.ratio | p.value |
|  | loud:cat | 1-2 | -10.554 | 2.39 | Inf | -4.421 | 0.0002 |
|  |  | 1-3 | -4.905 | 2.60 | Inf | -1.885 | 0.4901 |
|  |  | 1-4 | -7.133 | 2.59 | Inf | -2.759 | 0.0842 |
|  |  | 1-5 | 0.985 | 2.64 | Inf | 0.373 | 0.9998 |
|  |  | 1-6 | -5.621 | 2.34 | Inf | -2.399 | 0.1987 |
|  |  | 1-7 | -10.263 | 2.35 | Inf | -4.362 | 0.0003 |
|  |  | 2-3 | 5.649 | 2.34 | Inf | 2.419 | 0.1906 |
|  |  | 2-4 | 3.421 | 2.32 | Inf | 1.476 | 0.7593 |
|  |  | 2-5 | 11.540 | 2.38 | Inf | 4.855 | <.0001 |
|  |  | 2-6 | 4.934 | 2.04 | Inf | 2.415 | 0.1920 |
|  |  | 2-7 | 0.291 | 2.05 | Inf | 0.142 | 1.0000 |
|  |  | 3-4 | -2.228 | 2.54 | Inf | -0.877 | 0.9760 |
|  |  | 3-5 | 5.891 | 2.59 | Inf | 2.271 | 0.2581 |
|  |  | 3-6 | -0.716 | 2.29 | Inf | -0.312 | 0.9999 |
|  |  | 3-7 | -5.358 | 2.30 | Inf | -2.327 | 0.2310 |
|  |  | 4-5 | 8.118 | 2.58 | Inf | 3.147 | 0.0275 |
|  |  | 4-6 | 1.512 | 2.27 | Inf | 0.665 | 0.9944 |
|  |  | 4-7 | -3.130 | 2.28 | Inf | -1.372 | 0.8171 |
|  |  | 5-6 | -6.606 | 2.33 | Inf | -2.832 | 0.0693 |
|  |  | 5-7 | -11.248 | 2.35 | Inf | -4.796 | <.0001 |
|  |  | 6-7 | -4.642 | 2.00 | Inf | -2.315 | 0.2364 |
|  |  | contrast | estimate | SE | df | z.ratio | p.value |
|  | f0:lang | English - Hungarian | 1.53 | 0.513 | Inf | 2.988 | 0.0028 |
|  |  | contrast | estimate | SE | df | z.ratio | p.value |
|  | cl:lang | English - Hungarian | 4.04 | 0.519 | Inf | 7.792 | <.0001 |

*Table S1*

*Tukey post-hoc tests for the linear mixed model results of the intensity ratings, with pairwise comparisons in case of the categories and language, and trend comparisons in case of call length and categories, fundamental frequency and categories, loudness and categories, fundamental frequency and language, and call length and language. Cat: category, cl: call length, f0: fundamental frequency, lang: language of the query, loud: loudness of sound samples*

Intensity – estimates

Table S2

|  |  | Language | emmean | SE | df | asymp.LCL | asymp.UCL |
| --- | --- | --- | --- | --- | --- | --- | --- |
| lang\|cat | cat = 1 | English | 42.4 | 1.54 | Inf | 39.4 | 45.4 |
|  |  | Hungarian | 36.8 | 2.52 | Inf | 31.9 | 41.8 |
|  | cat = 2 | English | 72.9 | 3.03 | Inf | 66.9 | 78.8 |
|  |  | Hungarian | 60.7 | 3.66 | Inf | 53.5 | 67.8 |
|  | cat = 3 | English | 41.4 | 1.53 | Inf | 38.4 | 44.3 |
|  |  | Hungarian | 39.1 | 2.54 | Inf | 34.1 | 44.1 |
|  | cat = 4 | English | 46.1 | 1.53 | Inf | 43.1 | 49.1 |
|  |  | Hungarian | 46.1 | 2.54 | Inf | 41.1 | 51.1 |
|  | cat = 5 | English | 41.5 | 1.50 | Inf | 38.5 | 44.4 |
|  |  | Hungarian | 35.2 | 2.50 | Inf | 30.3 | 40.1 |
|  | cat = 6 | English | 54.9 | 1.35 | Inf | 52.3 | 57.6 |
|  |  | Hungarian | 47.3 | 2.41 | Inf | 42.6 | 52.0 |
|  | cat = 7 | English | 60.0 | 1.35 | Inf | 57.4 | 62.7 |
|  |  | Hungarian | 50.7 | 2.42 | Inf | 46.0 | 55.5 |
|  |  | Categories | emmean | SE | df | asymp.LCL | asymp.UCL |
| cat\|lang | English | 1 | 42.4 | 1.54 | Inf | 39.4 | 45.4 |
|  |  | 2 | 72.9 | 3.03 | Inf | 66.9 | 78.8 |
|  |  | 3 | 41.4 | 1.53 | Inf | 38.4 | 44.3 |
|  |  | 4 | 46.1 | 1.53 | Inf | 43.1 | 49.1 |
|  |  | 5 | 41.5 | 1.50 | Inf | 38.5 | 44.4 |
|  |  | 6 | 54.9 | 1.35 | Inf | 52.3 | 57.6 |
|  |  | 7 | 60.0 | 1.35 | Inf | 57.4 | 62.7 |
|  | Hungarian | 1 | 36.8 | 2.52 | Inf | 31.9 | 41.8 |
|  |  | 2 | 60.7 | 3.66 | Inf | 53.5 | 67.8 |
|  |  | 3 | 39.1 | 2.54 | Inf | 34.1 | 44.1 |
|  |  | 4 | 46.1 | 2.54 | Inf | 41.1 | 51.1 |
|  |  | 5 | 35.2 | 2.50 | Inf | 30.3 | 40.1 |
|  |  | 6 | 47.3 | 2.41 | Inf | 42.6 | 52.0 |
|  |  | 7 | 50.7 | 2.42 | Inf | 46.0 | 55.5 |
|  |  | Categories | cl.trend | SE | df | asymp.LCL | asymp.UCL |
|  | cl:cat | 1 | -1.482 | 0.936 | Inf | -3.32 | 0.352 |
|  |  | 2 | -2.437 | 0.866 | Inf | -4.13 | -0.741 |
|  |  | 3 | -5.317 | 0.946 | Inf | -7.17 | -3.463 |
|  |  | 4 | -1.709 | 0.924 | Inf | -3.52 | 0.102 |
|  |  | 5 | -0.177 | 0.930 | Inf | -2.00 | 1.645 |
|  |  | 6 | -1.095 | 0.809 | Inf | -2.68 | 0.491 |
|  |  | 7 | -0.388 | 0.849 | Inf | -2.05 | 1.275 |
|  |  | Categories | f0.trend | SE | df | asymp.LCL | asymp.UCL |
|  | f0:cat | 1 | 9.109 | 0.673 | Inf | 7.79 | 10.43 |
|  |  | 2 | 0.563 | 1.240 | Inf | -1.87 | 2.99 |
|  |  | 3 | 10.082 | 0.677 | Inf | 8.76 | 11.41 |
|  |  | 4 | 11.287 | 0.679 | Inf | 9.96 | 12.62 |
|  |  | 5 | 9.377 | 0.681 | Inf | 8.04 | 10.71 |
|  |  | 6 | 5.669 | 0.946 | Inf | 3.81 | 7.52 |
|  |  | 7 | 4.127 | 0.937 | Inf | 2.29 | 5.96 |
|  |  | Categories | loud.trend | SE | df | asymp.LCL | asymp.UCL |
|  | loud:cat | 1 | -1.23 | 1.87 | Inf | -4.903 | 2.44 |
|  |  | 2 | 9.32 | 1.48 | Inf | 6.420 | 12.22 |
|  |  | 3 | 3.67 | 1.81 | Inf | 0.129 | 7.21 |
|  |  | 4 | 5.90 | 1.78 | Inf | 2.403 | 9.39 |
|  |  | 5 | -2.22 | 1.86 | Inf | -5.867 | 1.43 |
|  |  | 6 | 4.39 | 1.41 | Inf | 1.628 | 7.15 |
|  |  | 7 | 9.03 | 1.43 | Inf | 6.233 | 11.82 |
|  |  | lang | f0.trend | SE | df | asymp.LCL | asymp.UCL |
|  | f0:lang | English | 7.94 | 0.335 | Inf | 7.28 | 8.60 |
|  |  | Hungarian | 6.41 | 0.519 | Inf | 5.39 | 7.42 |
|  |  | lang | cl.trend | SE | df | asymp.LCL | asymp.UCL |
|  | cl:lang | English | 0.221 | 0.348 | Inf | -0.462 | 0.903 |
|  |  | Hungarian | -3.822 | 0.534 | Inf | -4.869 | -2.776 |

*Table S2*

*Estimates for the linear mixed model results of the intensity ratings. Cat: category, cl: call length, f0: fundamental frequency, lang: language of the query, loud: loudness of sound samples.*

Valence - Tukey post-hoc tests

Table S3

|  | categories | contrast | estimate | SE | df | z.ratio | p.value |
| --- | --- | --- | --- | --- | --- | --- | --- |
| lang\|cat | cat = 1 | English - Hungarian | 0.589 | 2.41 | Inf | 0.244 | 0.8070 |
|  | cat = 2 |  | -5.386 | 2.39 | Inf | -2.255 | 0.0241 |
|  | cat = 3 |  | 5.998 | 2.43 | Inf | 2.473 | 0.0134 |
|  | cat = 4 |  | -0.574 | 2.43 | Inf | -0.236 | 0.8131 |
|  | cat = 5 |  | 2.074 | 2.41 | Inf | 0.859 | 0.3901 |
|  | cat = 6 |  | -2.732 | 2.41 | Inf | -1.134 | 0.2568 |
|  | cat = 7 |  | -2.148 | 2.41 | Inf | -0.890 | 0.3736 |
|  |  | contrast: categories | estimate | SE | df | z.ratio | p.value |
| cat\|lang | English | 1-2 | 17.524 | 2.12 | Inf | 8.263 | <.0001 |
|  |  | 1-3 | -2.205 | 1.53 | Inf | -1.438 | 0.7810 |
|  |  | 1-4 | -6.233 | 1.54 | Inf | -4.059 | 0.0010 |
|  |  | 1-5 | -4.220 | 1.51 | Inf | -2.797 | 0.0761 |
|  |  | 1-6 | 13.975 | 1.33 | Inf | 10.522 | <.0001 |
|  |  | 1-7 | 11.169 | 1.33 | Inf | 8.420 | <.0001 |
|  |  | 2-3 | -19.729 | 2.11 | Inf | -9.341 | <.0001 |
|  |  | 2-4 | -23.757 | 2.12 | Inf | -11.224 | <.0001 |
|  |  | 2-5 | -21.744 | 2.10 | Inf | -10.347 | <.0001 |
|  |  | 2-6 | -3.549 | 1.98 | Inf | -1.792 | 0.5536 |
|  |  | 2-7 | -6.355 | 1.98 | Inf | -3.214 | 0.0223 |
|  |  | 3-4 | -4.028 | 1.52 | Inf | -2.645 | 0.1127 |
|  |  | 3-5 | -2.015 | 1.49 | Inf | -1.348 | 0.8290 |
|  |  | 3-6 | 16.180 | 1.31 | Inf | 12.328 | <.0001 |
|  |  | 3-7 | 13.374 | 1.31 | Inf | 10.201 | <.0001 |
|  |  | 4-5 | 2.013 | 1.50 | Inf | 1.344 | 0.8312 |
|  |  | 4-6 | 20.208 | 1.32 | Inf | 15.365 | <.0001 |
|  |  | 4-7 | 17.402 | 1.31 | Inf | 13.250 | <.0001 |
|  |  | 5-6 | 18.195 | 1.28 | Inf | 14.188 | <.0001 |
|  |  | 5-7 | 15.389 | 1.28 | Inf | 12.016 | <.0001 |
|  |  | 6-7 | -2.806 | 1.06 | Inf | -2.643 | 0.1133 |
|  | Hungarian | 1-2 | 11.549 | 2.53 | Inf | 4.563 | 0.0001 |
|  |  | 1-3 | 3.205 | 2.06 | Inf | 1.556 | 0.7107 |
|  |  | 1-4 | -7.395 | 2.06 | Inf | -3.595 | 0.0060 |
|  |  | 1-5 | -2.735 | 2.03 | Inf | -1.350 | 0.8279 |
|  |  | 1-6 | 10.654 | 1.89 | Inf | 5.641 | <.0001 |
|  |  | 1-7 | 8.432 | 1.89 | Inf | 4.453 | 0.0002 |
|  |  | 2-3 | -8.344 | 2.53 | Inf | -3.294 | 0.0171 |
|  |  | 2-4 | -18.945 | 2.53 | Inf | -7.492 | <.0001 |
|  |  | 2-5 | -14.284 | 2.51 | Inf | -5.689 | <.0001 |
|  |  | 2-6 | -0.895 | 2.40 | Inf | -0.373 | 0.9998 |
|  |  | 2-7 | -3.117 | 2.41 | Inf | -1.295 | 0.8545 |
|  |  | 3-4 | -10.600 | 2.06 | Inf | -5.157 | <.0001 |
|  |  | 3-5 | -5.940 | 2.02 | Inf | -2.936 | 0.0517 |
|  |  | 3-6 | 7.449 | 1.89 | Inf | 3.947 | 0.0015 |
|  |  | 3-7 | 5.227 | 1.89 | Inf | 2.763 | 0.0834 |
|  |  | 4-5 | 4.660 | 2.02 | Inf | 2.310 | 0.2392 |
|  |  | 4-6 | 18.049 | 1.88 | Inf | 9.589 | <.0001 |
|  |  | 4-7 | 15.827 | 1.89 | Inf | 8.390 | <.0001 |
|  |  | 5-6 | 13.389 | 1.85 | Inf | 7.246 | <.0001 |
|  |  | 5-7 | 11.167 | 1.85 | Inf | 6.031 | <.0001 |
|  |  | 6-7 | -2.222 | 1.70 | Inf | -1.305 | 0.8500 |
|  |  | contrast: categories | estimate | SE | df | z.ratio | p.value |
|  | cl:cat | 1-2 | 3.013 | 1.26 | Inf | 2.400 | 0.1985 |
|  |  | 1-3 | 0.414 | 1.40 | Inf | 0.296 | 0.9999 |
|  |  | 1-4 | -2.688 | 1.38 | Inf | -1.942 | 0.4520 |
|  |  | 1-5 | 0.981 | 1.39 | Inf | 0.708 | 0.9922 |
|  |  | 1-6 | 4.002 | 1.26 | Inf | 3.177 | 0.0251 |
|  |  | 1-7 | 3.262 | 1.27 | Inf | 2.559 | 0.1388 |
|  |  | 2-3 | -2.599 | 1.26 | Inf | -2.066 | 0.3731 |
|  |  | 2-4 | -5.701 | 1.25 | Inf | -4.575 | 0.0001 |
|  |  | 2-5 | -2.031 | 1.24 | Inf | -1.632 | 0.6613 |
|  |  | 2-6 | 0.989 | 1.10 | Inf | 0.903 | 0.9722 |
|  |  | 2-7 | 0.249 | 1.11 | Inf | 0.224 | 1.0000 |
|  |  | 3-4 | -3.102 | 1.39 | Inf | -2.235 | 0.2766 |
|  |  | 3-5 | 0.568 | 1.39 | Inf | 0.409 | 0.9996 |
|  |  | 3-6 | 3.588 | 1.26 | Inf | 2.843 | 0.0673 |
|  |  | 3-7 | 2.848 | 1.28 | Inf | 2.230 | 0.2793 |
|  |  | 4-5 | 3.670 | 1.38 | Inf | 2.663 | 0.1078 |
|  |  | 4-6 | 6.690 | 1.25 | Inf | 5.350 | <.0001 |
|  |  | 4-7 | 5.950 | 1.27 | Inf | 4.702 | 0.0001 |
|  |  | 5-6 | 3.021 | 1.25 | Inf | 2.417 | 0.1913 |
|  |  | 5-7 | 2.281 | 1.26 | Inf | 1.803 | 0.5457 |
|  |  | 6-7 | -0.740 | 1.12 | Inf | -0.663 | 0.9945 |
|  |  | contrast: categories | estimate | SE | df | z.ratio | p.value |
|  | loud:cat | 1-2 | -4.014 | 2.19 | Inf | -1.837 | 0.5229 |
|  |  | 1-3 | 8.977 | 2.77 | Inf | 3.242 | 0.0203 |
|  |  | 1-4 | 11.847 | 2.75 | Inf | 4.310 | 0.0003 |
|  |  | 1-5 | 0.848 | 2.80 | Inf | 0.303 | 0.9999 |
|  |  | 1-6 | -0.881 | 2.28 | Inf | -0.387 | 0.9997 |
|  |  | 1-7 | 1.422 | 2.29 | Inf | 0.622 | 0.9961 |
|  |  | 2-3 | 12.991 | 2.13 | Inf | 6.103 | <.0001 |
|  |  | 2-4 | 15.861 | 2.10 | Inf | 7.546 | <.0001 |
|  |  | 2-5 | 4.862 | 2.16 | Inf | 2.254 | 0.2668 |
|  |  | 2-6 | 3.132 | 1.36 | Inf | 2.304 | 0.2421 |
|  |  | 2-7 | 5.436 | 1.37 | Inf | 3.956 | 0.0015 |
|  |  | 3-4 | 2.870 | 2.71 | Inf | 1.060 | 0.9396 |
|  |  | 3-5 | -8.129 | 2.76 | Inf | -2.949 | 0.0499 |
|  |  | 3-6 | -9.859 | 2.22 | Inf | -4.431 | 0.0002 |
|  |  | 3-7 | -7.555 | 2.24 | Inf | -3.379 | 0.0129 |
|  |  | 4-5 | -10.999 | 2.74 | Inf | -4.018 | 0.0012 |
|  |  | 4-6 | -12.728 | 2.20 | Inf | -5.790 | <.0001 |
|  |  | 4-7 | -10.425 | 2.21 | Inf | -4.724 | <.0001 |
|  |  | 5-6 | -1.729 | 2.25 | Inf | -0.768 | 0.9879 |
|  |  | 5-7 | 0.574 | 2.26 | Inf | 0.254 | 1.0000 |
|  |  | 6-7 | 2.304 | 1.52 | Inf | 1.520 | 0.7328 |

*Table S3*

*Tukey post-hoc tests for the linear mixed model results of the valence ratings, with pairwise comparisons in case of the categories and language, and trend comparisons in case of call length and categories, and loudness and categories. Cat: category, cl: call length, lang: language of the query, loud: loudness of sound samples*

Valence – estimates

Table S4

|  |  | Language | emmean | SE | df | asymp.LCL | asymp.UCL |
| --- | --- | --- | --- | --- | --- | --- | --- |
| lang\|cat | cat = 1 | English | -5.820 | 1.49 | Inf | -8.74 | -2.903 |
|  |  | Hungarian | -6.409 | 2.32 | Inf | -10.96 | -1.856 |
|  | cat = 2 | English | -23.344 | 2.08 | Inf | -27.42 | -19.267 |
|  |  | Hungarian | -17.958 | 2.74 | Inf | -23.34 | -12.579 |
|  | cat = 3 | English | -3.615 | 1.47 | Inf | -6.50 | -0.726 |
|  |  | Hungarian | -9.614 | 2.34 | Inf | -14.19 | -5.037 |
|  | cat = 4 | English | 0.412 | 1.48 | Inf | -2.48 | 3.310 |
|  |  | Hungarian | 0.986 | 2.33 | Inf | -3.58 | 5.555 |
|  | cat = 5 | English | -1.600 | 1.45 | Inf | -4.44 | 1.235 |
|  |  | Hungarian | -3.674 | 2.30 | Inf | -8.17 | 0.826 |
|  | cat = 6 | English | -19.795 | 1.26 | Inf | -22.26 | -17.326 |
|  |  | Hungarian | -17.063 | 2.18 | Inf | -21.33 | -12.792 |
|  | cat = 7 | English | -16.989 | 1.26 | Inf | -19.45 | -14.526 |
|  |  | Hungarian | -14.841 | 2.19 | Inf | -19.13 | -10.556 |
|  |  | Categories | emmean | SE | df | asymp.LCL | asymp.UCL |
| cat\|lang | English | 1 | -5.820 | 1.49 | Inf | -8.74 | -2.903 |
|  |  | 2 | -23.344 | 2.08 | Inf | -27.42 | -19.267 |
|  |  | 3 | -3.615 | 1.47 | Inf | -6.50 | -0.726 |
|  |  | 4 | 0.412 | 1.48 | Inf | -2.48 | 3.310 |
|  |  | 5 | -1.600 | 1.45 | Inf | -4.44 | 1.235 |
|  |  | 6 | -19.795 | 1.26 | Inf | -22.26 | -17.326 |
|  |  | 7 | -16.989 | 1.26 | Inf | -19.45 | -14.526 |
|  | Hungarian | 1 | -6.409 | 2.32 | Inf | -10.96 | -1.856 |
|  |  | 2 | -17.958 | 2.74 | Inf | -23.34 | -12.579 |
|  |  | 3 | -9.614 | 2.34 | Inf | -14.19 | -5.037 |
|  |  | 4 | 0.986 | 2.33 | Inf | -3.58 | 5.555 |
|  |  | 5 | -3.674 | 2.30 | Inf | -8.17 | 0.826 |
|  |  | 6 | -17.063 | 2.18 | Inf | -21.33 | -12.792 |
|  |  | 7 | -14.841 | 2.19 | Inf | -19.13 | -10.556 |
|  |  | Categories | cl.trend | SE | df | asymp.LCL | asymp.UCL |
|  | cl:cat | 1 | -1.26 | 0.984 | Inf | -3.188 | 0.671 |
|  |  | 2 | -4.27 | 0.778 | Inf | -5.797 | -2.746 |
|  |  | 3 | -1.67 | 0.989 | Inf | -3.610 | 0.266 |
|  |  | 4 | 1.43 | 0.974 | Inf | -0.478 | 3.338 |
|  |  | 5 | -2.24 | 0.975 | Inf | -4.150 | -0.330 |
|  |  | 6 | -5.26 | 0.785 | Inf | -6.799 | -3.721 |
|  |  | 7 | -4.52 | 0.809 | Inf | -6.106 | -2.935 |
|  |  | Categories | loud.trend | SE | df | asymp.LCL | asymp.UCL |
|  | loud:cat | 1 | -5.96 | 1.988 | Inf | -9.85 | -2.061 |
|  |  | 2 | -1.94 | 0.903 | Inf | -3.71 | -0.175 |
|  |  | 3 | -14.93 | 1.928 | Inf | -18.71 | -11.156 |
|  |  | 4 | -17.80 | 1.899 | Inf | -21.53 | -14.082 |
|  |  | 5 | -6.81 | 1.971 | Inf | -10.67 | -2.943 |
|  |  | 6 | -5.08 | 1.111 | Inf | -7.25 | -2.899 |
|  |  | 7 | -7.38 | 1.130 | Inf | -9.59 | -5.165 |

*Table S4*

*Estimates for the linear mixed model results of the valence ratings. Cat: category, cl: call length, lang: language of the query, loud: loudness of sound samples.*
